# Detecting Peripheral Neuropathy in Patients with Diabetes, Prediabetes and other High-Risk Conditions: An Advanced Practice Nurse’s Perspective

**DOI:** 10.47363/jmcn/2022(3)143

**Published:** 2022-03-21

**Authors:** Joyce K Anastasi, Bernadette Capili

**Affiliations:** 1 Special Studies in Symptom Management New York University, New York, USA; 2 Heilbrunn Family Center for Research Nursing, New York, USA

**Keywords:** Peripheral Neuropathy, Neuropathy Detection, Patient-Focused, QOL, Diabetic Neuropathy, HIV Neuropathy

## Abstract

A common complication of diabetes, HIV infection, and other chronic systemic conditions and exposures, distal sensory peripheral neuropathy is increasingly prevalent worldwide; the physical, mental, and economic burdens are significant. As no curative therapies exist to date, early detection of peripheral neuropathy (PN) affords patients the best chance to reverse it through education, intensive lifestyle modifications, and multidisciplinary management. Concerning diabetic PN, obstacles to effective screening include low clinical priority, failure to screen patients during prediabetes, confusion regarding methods and goals of testing, and possibly inexperience with thermal testing. Providers and advanced practice nurses are well-positioned to advocate for and implement early PN detection programs, screen for complications including sleep and mood disorders, promote multidisciplinary management, identify strategies to reduce pain and other PN symptoms, and counsel patients regarding many aspects of safety and self-care for improved quality of life. This manuscript provides a brief overview of PN with an emphasis on diabetic PN, a discussion of the aforementioned obstacles to effective screening, and a summary of recommendations to improve PN identification in clinical practice.

## Background

### Sensory Peripheral Neuropathies

Diabetes mellitus (DM) is estimated to affect greater than 37 million individuals in the US-roughly 11% of the population-and greater than 400 million individuals worldwide [1,2]. An estimated one quarter of US adults with DM are undiagnosed. Another 96 million, a staggering 38% of US adults over 18, are thought to have prediabetes (also called borderline diabetes or glucose intolerance) [1]. As population demographics tip increasingly toward older age groups and as sedentary lifestyles persist, the prevalence of DM is expected to keep rising, surpassing 600 million globally by 2045 [2].

As is well known, DM may affect any organ in the body, including the heart, eyes, kidneys, and nervous system, with risks for complications increases with disease duration. By 10 years into the diagnosis, at least 50% of type 2 DM patients develop some form of neuropathy, the vast majority being a type of peripheral neuropathy known as distal symmetric polyneuropathy (DSPN) or diabetic peripheral neuropathy (DPN) [3,4].

Diabetic PN characteristically affects multiple somatosensory nerves of the feet and lower limbs and sometimes the hands and upper extremities in a bilateral (or “symmetric”) distribution [5]. Estimates suggest that DPN represents between 75% and 90% of PN cases among patients with DM and 10% to 30% of patients with prediabetes; thus, based on the most current prevalence data from the CDC, between 23 and 45 million individuals suffer from DPN in the US alone [1,3,4].

By definition, peripheral neuropathies involve damage to nerves that carry signals back and forth between the central nervous system (brain and spinal cord) and the periphery (skin, muscles, and end-organs). The pathogenesis is often complex and may result from one or more insults that may be metabolic, vascular, toxic, nutritional, infectious, inflammatory, traumatic, and hereditary [3,6]. For example, DPN is thought to represent the cumulative effects of oxidative stress and inflammation from diabetes-induced metabolic imbalances, ie., hyperglycemia, insulin resistance, hyperlipidemia, and protein catabolism, leading to microvascular and macrovascular disease. These factors first injure the nerves that mediate temperature and pain perception (mostly small unmyelinated and thinly myelinated C fibers), then involve larger nerve fibers responsible for mediating vibration, proprioception, and light touch [3,7]. In addition to DM, common causes of sensory PN include HIV-associated sensory neuropathy (also called HIV-distal sensory peripheral neuropathy or HIV-DSP), which affects up to 50% of persons living with HIV (PLWH), and chemotherapy-induced peripheral neurotoxicity (CIPN), which affects 30-40% of patients exposed to specific anti-neoplastic agents including platinum compounds, taxanes and vinca alkaloids [4,5,8-10]. Other infections, toxic exposures, liver or kidney disease, nutritional deficiencies, alcohol consumption or autoimmune-mediated inflammation may also contribute to the development of PN [4,5].

Thirty percent to 40% of PN cases are idiopathic, ie., not linked to any discernible underlying disease, although some studies have shown higher than average rates of impaired glucose tolerance (IGT) and dyslipidemia among patients with idiopathic PN, which may be contributing factors [6,11-13].

### A Heavy Burden

Distal sensory peripheral neuropathy (DSPN) is associated with physical, psychosocial, and financial burdens to patients that are sometimes mild but may be severe and debilitating [14]. Patients commonly complain of tingling, stabbing, electric sensations, pain in the feet, or numbness. Pain may be worse at night and aggravated by the touch of clothing and bedsheets (allodynia), interfering with the ability to sleep at night or concentrate during the day. Painful DSPN-present in 20% to 40% of patients with DPN-is associated with reduced activity and mobility, increased mortality, and greatly increased financial costs [6,15-17].

Pain and other symptoms take a tremendous toll on PN patients’ quality of life (QOL) and functionality, interfering with activities of daily living and contributing to anxiety, depression, and a loss of personal agency [7,14]. Patients with PN, in particular those with loss of balance and sensation, are at an increased risk for injury from falls, bums, and secondary infections should a foot wound go unnoticed due to lack of pain or “loss of protective function” (LOPF) [18].

When compounded by peripheral artery disease and infection, DPN may lead to diabetic foot disease and ulceration, a harrowing complication experienced by 1 in 4 DM patients in the course of their illness. Notoriously difficult to treat, foot ulceration may progress to the point of needing limb amputation, significantly increasing all of the burdens described above as well as patient mortality [19].

Globally, expenditures related to DM were estimated at 375 billion US dollars in 2010, and projected to top 490 billion by 2030, in part due to urbanization and rising DM incidence in China and India [20,21]. And that may be a vast underestimate, as annual expenditures in the US alone were reported at 245 billion in 2012, 27% of which attributable to neuropathy [17]. The US spends roughly 12% of its healthcare budget on DM, and loses 3.6 billion dollars in revenue annually from work lost related to DM-related disabilities, including DPN, annually [20,22]. The development of DPN doubles patients’ healthcare costs; the development of painful DPN quadruples costs [17].

### Detecting PN

If we should accept that DPN rates and associated burdens and costs-already alarmingly high-are steadily rising, and that detecting DPN early reduces patients’ risk for poor outcomes (see lifestyle and exercise discussion below), it is worthwhile to wonder, why is there not greater urgency around DPN screening? And what other issues or misunderstandings might be impeding efforts to detect and diagnose DPN? [7].

### Be Proactive

To the first question, there may understandably exist a disinterest, lack of motivation, even a malaise, among providers to screen for a condition they perceive to be untreatable and for which the prognosis is frustratingly poor [23]. It is not uncommon for providers (and hospitals and insurance providers) to equate a lack of pharmaceutical treatment with an inability to help and/or to significantly affect the course of a disease. While it is true that DPN is not immediately life-threatening, and there are currently no FDA-approved disease-modifying treatments (except oral glucose-lowering agents, insulin, and medications such as pregabalin for neuropathic pain), and while it is always advisable to manage patient expectations wisely, an attitude of nihilism around PN is unhelpful to patients, as it may contribute to depressed mood thus potentially interfering with self-care and adherence to treatments [24,25].

Moreover, nonpharmaceutical interventions may be of use to patients. There is evidence, for example, that exercise, dietary change, and weight loss may alter the natural history of prediabetes, diabetes, prediabetes-associated neuropathy, and DPN [16,26-29]. The large, prospective Nurses’ Health Study from the 1990s showed that women who exercised regularly-whether walking around the house for 2 hours or taking brisk walks outside for 1 hour-developed type 2 DM at significantly lower rates compared with women who did less exercise or spent that time watching TV [30]. In a seminal prospective trial published in the New England Journal of Medicine in 2002, patients with prediabetes who received dietary and exercise counseling with the goal of long-term body weight reduction of at least 7% were significantly less likely to develop type 2 DM compared matched subjects treated with metformin or placebo who did not receive the counseling [26].

Four years later. Smith and colleagues showed that patients with prediabetes-related neuropathy who underwent a similar intervention promoting dietary changes and exercise over one year experienced significantly reduced pain, and although not designed to show it, improved metabolic parameters of reduced BMI, oral glucose tolerance test (OGTT), and serum cholesterol [16]. The main endpoint of the study was improvement in intraepidermal nerve fiber density (IENFD) on skin biopsy, which was significantly increased at one year compared to baseline, indicating that metabolic improvements from a healthier diet and more exercise had the power to reverse nerve damage if implemented in prediabetes [16].

In a separate study, patients with types 1 or 2 DM without symptoms of DPN who participated in a moderate aerobic exercise training for 4 years were significantly less likely to develop DPN compared with matched controls who did not participate [27]. Several small studies have demonstrated improvements in symptoms, nerve conduction, and intraepidermal nerve brandling among patients with diabetes and DPN who participated in aerobic exercise programs [28,29]. These studies taken together, along with others that have correlated diminished sensory function (measured by quantitative sensory testing or QST) with progression from no DPN to painless DPN to painful DPN, point to something that should come as very good news for patients-DPN is at least partially reversible with intensive exercise and may be preventable if caught early in its course [16,27-31].

In patients with pain, complementary and alternative medicine (CAM) therapies may be used in patients who cannot tolerate standard therapies or used adjunctively to optimize pain management. Several have shown efficacy in the treatment of diabetic neuropathic pain, particularly topical capsaicin and acupuncture for which there is the largest body of evidence, but also transcutaneous electrical nerve stimulation (TENS) and supplementation with alpha lipoid acid and acetyl-1-carntine [24,32-34].

Providers reluctant to evalutate PN should remember that a diagnosis provides the basis for patients to obtain the educational materials, counseling and resources they will need to stay well, including information about lifestyle modification, glucose control, foot inspection, skincare, symptom management, infection prevention and treatment, fall prevention, and psychosocial health [25]. Patients aware of their diagnosis may choose to try experimental modalities CAM therapies or enroll in clinical trials, any or all of which may reduce symptoms and improve QOL.

### Screen Patients with Prediabetes

More than one-third of the adult population is in the stage of glucose dysregulation that precedes diabetes known as prediabetes, which may present as impaired glucose tolerance or impaired fasting glucose, and is a significant risk factor for PN [1]. Depending on the study method, between 10% and 77% of patients with prediabetes have PN, which may be subclinical or symptomatic [35]. Despite their high risk, the American Diabetes Association (ADA) recommends that PN screening be “considered” among patients with prediabetes and only when symptoms are present [3]. Thus, providers who do not screen their prediabetes patients for PN are in adherence with the recommendation, as are those who screen only symptomatic patients (which does little to boost detection rates as testing for hyperglycemia and glucose intolerance is already included in the work-up for symptomatic PN).

Based on current ADA guidance, which is not well concordant with PN epidemiology and pathogenesis, it is likely that millions of early PN cases among prediabetes patients are being missed. This is particularly unfortunate knowing that PN is most amenable to lifestyle interventions in its early stages (see above), after which it becomes increasingly difficult to treat [36]. A more successful (albeit labor-intensive) approach to PN screening might be treat prediabetes similar to diabetes, that is, perform a yearly history, exam, and sensory tests, including thermal testing or pinprick looking for small-fiber pathology that predominates in prediabetes PN [3,35].

### Screen for Small Fiber Disease

Experts debate the best bedside DPN screening method, as there is no gold standard test, particularly for detecting early disease [6]. The ADA recommends annual PN screening for all DM patients (beginning at the time of a type 2 DM diagnosis and 5 years following a type 1 DM diagnosis) by history, physical examination including ankle reflexes, either pinprick or temperature sensation (to assess small fiber disease), vibration sensation with tuning fork (to assess large fiber disease), and 10g monofilament testing for sensitivity to light pressure to assess for LOPF and risk for foot ulceration [3]. Screening or composite questionnaires, such as the Michigan Neuropathy Screening Instrument, which combines screening questions, foot exam, and neurologic assessment, are widely used [37].

The annual foot exam recommended by The International Working Group for the Diabetic Foot (IWGDF) emphasizes large fiber assessments, including 10g monofilament and vibrational sensation, excluding assessments for small fiber neuropathy assessments [38]. Similarly, the International Diabetes Foundation (IDF) recommends type 2 DM patients be tested annually for light touch sensation by 10g monofilament solely [39]. In some studies, 10 g monofilament examination has been shown to differentiate DM patients with and without neuropathy, and, along with the 128 Hz tuning fork, are widely employed in routine clinical practice [40,41]. However, similar to the lack of a strong recommendation for PN testing among patients in prediabetes, relying solely upon methods that detect large fiber, ie., later stage or mixed type, PN is controversial, as it may contribute to an underdiagnosis of cases, especially those characterized by pure small fiber neuropathology and those in an early stages [37].

A reasonable strategy for detecting a greater proportion of DPN cases would be to comply with the ADA recommendation and perform multiple assessments at the annual screening of DM patients with or without symptoms, including at least one small fiber assessment, eg., pinprick or thermal testing [3,40,42,43]. Reliability of pinprick sensitivity testing was found lacking on one meta-analysis [44]. Thermal testing, on the other hand, has been shown to be sensitive to small changes indicative of subclinical small fiber neuropathy among DM patients and may be preferred [7,31,42,43]. A modified Neuropathy Disability Score (NDS), which includes pinprick, temperature, vibrational assessments and ankle reflexes, covers most of the ADA recommended tests and can be performed quickly at the bedside [45].

For a more comprehensive exploration of DPN diagnostic methodology, including advantages and limitations of bedside and confirmatory options which is beyond the scope of this article, the reader is referred to a recent review by Carmichael and colleagues [37].

### Stepping into The Center

Advanced practice nurses are well positioned to take the lead in overcoming detection reluctance and advocating for and implementing broader in-office DPN and PN screening for at-risk patients. We can ally with patients by standing up for QOL; championing aggressive symptom management including pain management; educating patients regarding proper foot and skin care, DPN prevention, fall prevention and other vital topics; and being a hub in the center of a multidisciplinary team [25].

Not an exhaustive list, the following are suggestions for PN and DPN screening and counseling:

- Patients at risk for prediabetes-including adults over 50 with overweight, obesity, smoking, hyperlipidemia, hypertension, sedentary lifestyle or standard Western diet-should be counseled regarding lifestyle changes to reduce their risk for developing diabetes and heart disease
- Patients with documented prediabetes should also be counseled about lifestyle and diet modification, and should undergo regular screening for DPN [3].
- Proper DPN screening for patients with prediabetes or diabetes should include history, screening questionnaire, physical and neurological exam of the extremities (including ankle reflexes), and bedside peripheral neurologic testing including temperature sensation or pinprick (small fiber) and vibration, light touch (via 10g monofilament), and/or proprioception (large fiber) [3].
- Patients with idiopathic PN should be assessed for IGT with OGTT, fasting plasma glucose, and serum hemoglobin A1c, and dyslipidemia by serum lipid panel [12,13].
- Overweight patients with idiopathic PN should be counseled regarding diet, exercise, and weight loss [13].
- Patients with sensory neuropathy of any kind should be screened for anxiety, depression, and insomnia and offered treatment or specialist referral [46].
- Patients with painful PN should be evaluated for psychotropic agents and/or experimental therapies [24,47,48].
- Patients with DPN and at-risk for DPN should be counseled regarding lifestyle modifications and practices to minimize risk for complications, including reducing sedentary activities; improving diet and exercise; and smoking cessation [21,30]
- Patients with DPN at risk for diabetic foot disease should be counseled regarding foot inspection and care; appropriate footwear; and prevention of bums, falls and infections [19].

## Conclusion

As research in our understanding of PN evolves, it is helpful to shift focus from what is missing-a cure-to the many ways at-risk and affected patients benefit from attentive, skillful, multifaceted care. Perhaps even more than highly treatable diseases, championing early PN detection, early intervention, and QOL help patients achieve an easier, more satisfying life, a worthy goal for advanced practice nurses and all involved providers.

## List of Abbreviations:

CIPN: chemotherapy induced peripheral neuropathy
DM: diabetes mellitus
DPN: diabetic peripheral neuropathy
DSPN: distal sensory peripheral neuropathy
HIV-DSP: HIV distal sensory peripheral neuropathy
PN: peripheral neuropathy
